# Significant infrarenal aortic stenosis in pregnancy: a case report

**DOI:** 10.1186/s13256-019-2057-0

**Published:** 2019-05-01

**Authors:** Edmund Yin Man Chung, Anushree Tiku, Sean Seeho, Amanda Mather

**Affiliations:** 1 Renal Department, Royal North Shore Hospital, St Leonards, Australia; 2 Northern Clinical School, The University of Sydney, Camperdown, Australia; 3 Renal Department, St George Hospital, Kogarah, Australia; 4 Faculty of Medicine and Health, The University of Sydney, Camperdown, Australia; 5 Clinical and Population Perinatal Health Research, Kolling Institute, St Leonards, Australia

**Keywords:** Coarctation of the aorta, Pregnancy, Hypertension

## Abstract

**Background:**

Hypertension is common in pregnant women presenting with aortic coarctation or Takayasu’s arteritis. Uncontrolled hypertension leads to increased adverse maternal and neonatal events.

**Case presentation:**

A 36-year-old gravida 2, para 1 Caucasian woman presented at 9 weeks of gestation with headaches but normal blood pressure. She had a past medical history of an *in vitro* fertilization pregnancy complicated by preeclampsia at 27 weeks of gestation (birth weight 1900 g) and infrarenal aortic stenosis. In the current pregnancy, she received aspirin and calcium as preeclampsia prophylaxis, remained normotensive throughout pregnancy, and was delivered by elective cesarean section at 37 weeks without complications.

**Conclusions:**

This case demonstrates a significant chronic aortopathy in pregnancy with normal fetal growth and uterine blood flow through collateral supply from the internal mammary and epigastric arteries.

**Electronic supplementary material:**

The online version of this article (https://doi.org/10.1186/s13256-019-2057-0) contains supplementary material, which is available to authorized users.

## Background

Abdominal aortic coarctation is rare, accounting for approximately 2% of all aortic stenoses [1]. The average age at diagnosis is 20.7 years, and it occurs equally in both sexes [1]. In a review of 146 patients with abdominal aortic coarctation, 49% of lesions were interrenal, 17% were suprarenal, 23% were infrarenal, and 11% were diffuse [1]. Identifying the cause of the abdominal aortic coarctation is often not possible because the end-stage clinical and pathologic changes are nonspecific [2].

Coarctation of the aorta [3] and Takayasu’s arteritis [4] share a similar risk of hypertension (26–30%), miscarriage (9%), and preterm delivery (3–8%). Infants of women with these conditions also have similar birth weights (3.2–3.5 kg). Although the risk of preeclampsia is similar in coarctation of the aorta and inactive Takayasu’s arteritis (2–5%), the risk of preeclampsia is much higher in active Takayasu’s arteritis (21%) [3, 4]. Coarctation of the aorta in pregnancy is associated with a high risk of aortic dissection and maternal mortality up to 4%, mainly related to aortic rupture or dissection in the setting of hypertension [5]. We present a case of a patient with an uncomplicated second pregnancy in the setting of significant infrarenal aortic stenosis, and we review the literature on interventions for aortopathies in pregnancy.

## Case presentation

**Fig. 1:**
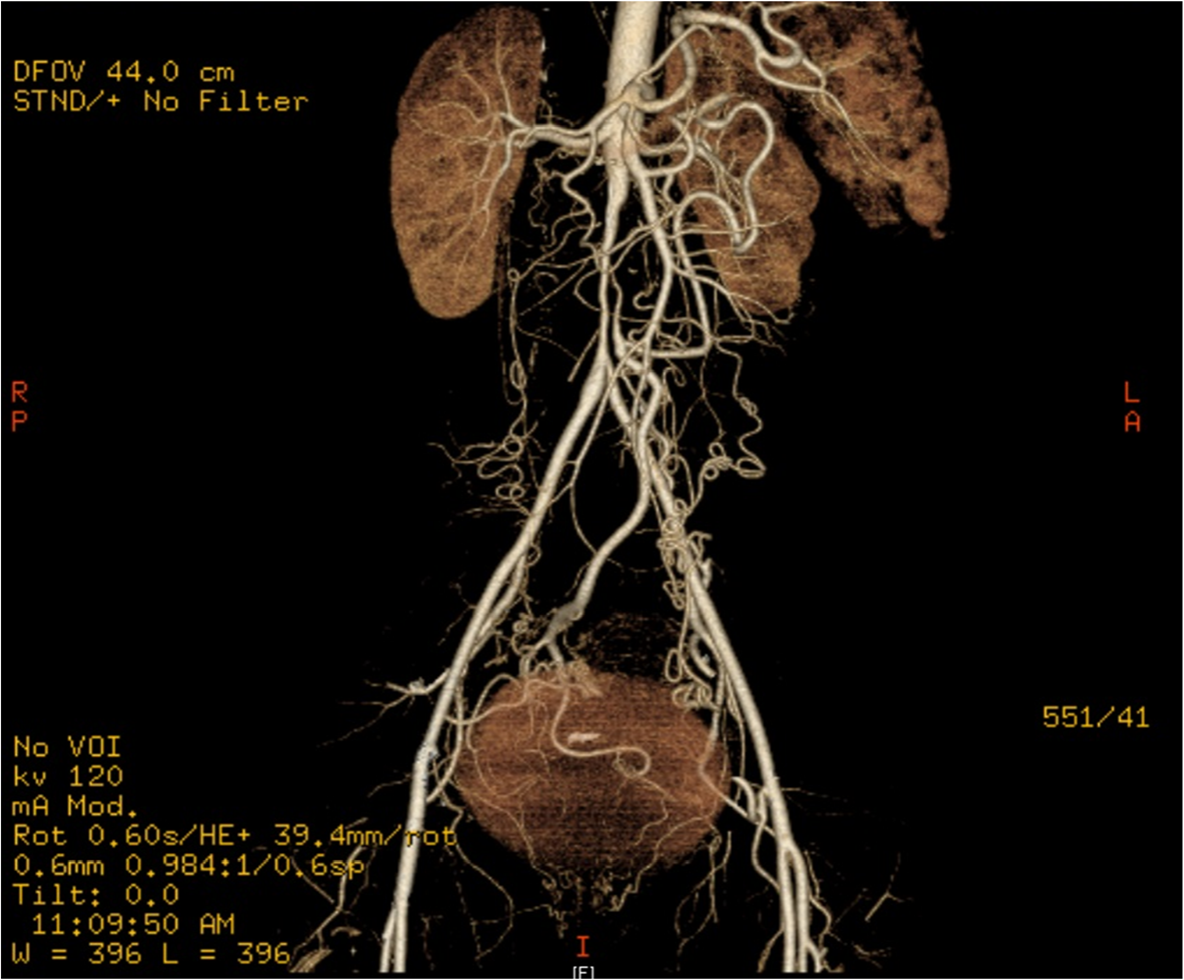
CT angiogram showing 75% infrarenal aortic stenosis with collateral circulation from the internal mammary and epigastric arteries

A 36-year-old gravida 2, para 1 Caucasian woman presented at 9 weeks of gestation with headaches. She was normotensive and had no visual changes, chest pain, dyspnea, or other neurological symptoms. Her previous *in vitro* fertilization pregnancy was complicated by preeclampsia at 27 weeks of gestation. A growth-restricted fetus was delivered by cesarean section at 36 weeks, weighing 1900 g Additional file 1. Other past history was significant for infrarenal aortic stenosis diagnosed on the basis of a computed tomographic angiogram obtained to investigate persistent hypertension and intermittent claudication postpartum, which showed 75% stenosis of the infrarenal aorta with hypertrophied internal mammary and epigastric arteries (Fig. 1). The remaining aortic branches were largely spared. Her regular medications were aspirin 100 mg and calcium 1.2 g daily. Her family history was significant for paternal ischemic heart disease and maternal hypertension.

Laboratory investigations for preeclampsia during her pregnancy revealed low-grade proteinuria (urine protein/creatinine ratio 40 mg/mmol) and normal renal and liver function. A vasculitic screen revealed a normal C-reactive protein (3.8 mg/L); mildly elevated erythrocyte sedimentation rate (ESR) (16 mm/h); and absence of antinuclear antibodies, antineutrophilic cytoplasmic antibodies, anti-double-stranded DNA antibodies, and antiphospholipid antibodies. The estimated fetal weight at a 34-week ultrasound was in the 94th percentile, and placental vascular resistance was normal.

The differential diagnoses for the major finding of significant infrarenal aortic stenosis included congenital abdominal coarctation, Takayasu’s arteritis, fibromuscular dysplasia, aortic neurofibromatosis, aortic tuberculosis, and radiation aortitis [1, 2, 6]. The presence of a well-developed collateral vasculature suggested a chronic aortopathy. In the absence of a clinical history or signs of neurofibromatosis, tuberculosis, or radiation exposure, as well as little evidence of active inflammation, the diagnosis of chronic abdominal aortopathy from congenital abdominal aortic coarctation, fibromuscular dysplasia, or inactive Takayasu’s arteritis was made.

The woman received aspirin and calcium as preeclampsia prophylaxis until 36 weeks of gestation, as well as insulin for gestational diabetes. She remained normotensive throughout pregnancy without requiring antihypertensive medications and delivered a healthy female infant weighing 3185 g by cesarean section at 37 weeks without complications. No regular medications were continued during the postpartum period.

## Discussion and conclusions

Abdominal aortic coarctation presents a significant risk of hypertension and aortic dissection in pregnancy. In our patient, the first pregnancy was complicated by preeclampsia and intrauterine growth restriction, but the second pregnancy was uncomplicated.

A case report in the literature described interrenal abdominal aortic coarctation with left renal artery stenosis and a right duplex system with fibromuscular dysplasia in a patient who had an initial pregnancy complicated by hypertension [7]. Left and right renal angioplasty were unsuccessful postpartum, and a left nephrectomy and right aortorenal bypass were performed with subsequent normalization of her blood pressure. A second pregnancy, with the patient receiving aspirin, was complicated by hypertension and premature delivery at 32 weeks of gestation due to the development of a false aneurysm of the aorta [7]. There are no other reports of abdominal aortic coarctation or aortic fibromuscular dysplasia in pregnancy.

Optimal management of aortic coarctation in pregnancy is unclear because there is a paucity of data in existing literature. Owing to the higher risk of aortic dissection and rupture in aortopathies during pregnancy, preconception management is critical to ensure a woman planning her pregnancy is counseled on the risk of aortic dissection, has a full cardiac assessment (including electrocardiogram, echocardiogram, and genetic consultation where relevant), and receives multidisciplinary care in a tertiary obstetric center to tailor management and monitoring to the patient and type of aortopathy [5]. In our patient, after the diagnosis of aortic coarctation, the second pregnancy was managed in a tertiary obstetric center with multidisciplinary care involving renal and surgical departments. The recommendation for serial echocardiograms is for surveillance of aortic root dilation [5], which was less relevant in our patient.

Evidence for surgical intervention for aortic coarctation before pregnancy is limited. Retrospective data from women with surgically treated coarctation of the aorta showed a 17% incidence of preeclampsia in subsequent pregnancies, which is higher than in other cohort studies [3, 4], though the sample size was small [8]. A larger retrospective study found no difference in hypertension, miscarriage, or birth weight between native coarctation and surgically repaired coarctation [3]. However, the strength of this conclusion is weakened by the retrospective nature of the data and associated selection bias. Despite the suggestion of stent placement or surgical repair of aortic coarctation in the management of uncontrolled hypertension during pregnancy [5], there are no studies or case reports examining the efficacy or safety of this approach. A case report of stent placement for aortic coarctation postpartum was successful with normal blood pressure at 18 months postpartum and no re-coarctation on echocardiography [9].

Evidence for the use of aspirin or calcium in aortic coarctation or Takayasu’s arteritis during pregnancy is lacking. In a retrospective study of pregnant women with Takayasu’s arteritis, aspirin prophylaxis was given in only 21% of cases, with no significant reduction in adverse events (odds ratio 1.23, 95% confidence interval 0.47–3.26); however, this study was not adequately powered to detect differences between the two groups [4]. In a retrospective study of pregnant Japanese women with Takayasu’s arteritis, only one woman took prophylactic aspirin, and she developed gestational hypertension [10]. In a case report of a 35-year-old gravida 2, para 1 woman with Takayasu’s arteritis involving the ascending and descending thoracic aorta treated with prophylactic aspirin and calcium, there were no adverse pregnancy outcomes [11]. Another case report involved a 25-year-old primigravida woman with active Takayasu’s arteritis involving the left subclavian and common carotid artery who was treated with prednisone and etanercept, as well as prophylactic aspirin for left arm claudication. Her first pregnancy was complicated by preeclampsia and spontaneous rupture of membranes, but her second pregnancy 8 months later, during which her ESR was improved but still elevated, was uncomplicated [12]. No case reports or case series of aortic coarctation during pregnancy documented use of aspirin or calcium.

We report a case of a patient with aortic coarctation detected due to persistent hypertension and intermittent claudication postpartum, demonstrating the necessity to evaluate causes of chronic hypertension in young patients. Clinicians should be aware that hypertension and aortic dissection are more frequent in women with aortopathies such as aortic coarctation, fibromuscular dysplasia, and Takayasu’s arteritis. Therefore, pregnant women with these comorbidities should be managed by a multidisciplinary team at a tertiary obstetric center. There is a lack of evidence to support the routine use of surgical interventions to improve neonatal or maternal outcomes or the use of aspirin or calcium as preeclampsia prophylaxis in the setting of aortic coarctation.

## Additional file

Additional file 1: Patient timeline. (DOC 29 kb)
