# Supplementary material for: Significant infrarenal aortic stenosis in pregnancy: a case report
Source: J Med Case Rep. 2019 May 1;13:115. doi: 10.1186/s13256-019-2057-0 (PMC6492387; doi:10.1186/s13256-019-2057-0)
Supplement: Supplementary file 1 — Patient timeline. (DOC 29 kb) [file 13256_2019_2057_MOESM1_ESM.doc]

Infrarenal aortic stenosis diagnosed on CT angiogram to investigate persistent hypertension and intermittent claudication postpartum, which showed 75% stenosis of the infrarenal aorta

First pregnancy through invitro fertilisation, complicated by pre-eclampsia at 27 weeks (intra-uterine growth restriction and hypertension), delivering via Caesarean-section at 36 weeks

Second pregnancy of natural conception. Received aspirin and calcium as pre-eclampsia prophylaxis. Developed gestational diabetes requiring insulin but remained normotensive and delivered a healthy female infant weighing 3185 grams by Caesarean-section at 37 weeks

2018

2013
